# The experience of intimate partner violence among older women: A narrative review

**DOI:** 10.1016/j.maturitas.2018.12.011

**Published:** 2019-03

**Authors:** Neha Pathak, Rageshri Dhairyawan, Shema Tariq

**Affiliations:** a UCL Research Department of Epidemiology & Public Health, Institute of Epidemiology & Healthcare, 1-19 Torrington Place, London, WC1E 6BT, UK; b UCL Institute of Health Informatics, 222 Euston Road, London, NW1 2DA, UK; c Barts Health NHS Trust, Royal London Hospital, Whitechapel Road, London, E1 4BB, UK; d UCL Institute for Global Health, Mortimer Market Centre, Off Capper Street, London, WC1E 6JB, UK

**Keywords:** Intimate partner violence, Domestic violence, Domestic abuse, Older women, Midlife

## Abstract

Intimate partner violence (IPV) against women is a significant public health issue globally. It has serious physical and psychological health consequences as well huge economic and social costs. With an ageing population globally, it is important to understand how older women experience IPV. We present a narrative review of 48 studies exploring IPV in women aged ≥45 years, focusing on: (1) prevalence of IPV; (2) factors associated with IPV; (3) impact of IPV; (4) responses to IPV; (5) IPV interventions; and (6) key populations. Although we found significant gaps in the literature and an inconsistency in definitions, data suggest that IPV is commonly experienced by older women (lifetime prevalence 16.5%–54.5%), but that their age and life transitions mean that they may experience abuse differently to younger women. They also face unique barriers to accessing help, such as disability and dependence on their partners. We recommend commissioning services that are specifically tailored to meet their needs. Professionals working in frontline services where older women are commonly seen should be trained to identify and respond to IPV appropriately.

## Highlights

• Older women’s experience of intimate partner violence (IPV) is an important public health concern.
• Globally, the reported prevalence of intimate partner violence experienced by older women ranges from 16.5% to 54.5%.
• The precipitants and consequences of intimate partner violence may be different for older than for younger women..
• Older women experiencing intimate partner violence face unique barriers to accessing help.
• Older women’s experience of intimate partner violence is under-researched, especially in terms of interventions.

## 1 Introduction

Violence against women (VAW) is a significant global public health issue and a fundamental violation of human rights [1]. A World Health Organisation multi-country study measured lifetime prevalence of physical and/or sexual partner violence at 15%–71% [2]. Intimate partner violence (IPV) is physical, sexual or psychological violence by a current or former partner or spouse [3] and is associated with enormous social and economic costs [4,5]. IPV can lead to multiple short and long-term physical and psychological sequelae including: traumatic injury, chronic pain, gastrointestinal disorders, depression, substance abuse, sexually transmitted infections and poor reproductive health [6,7]. Policies addressing IPV and VAW are therefore an important priority globally [8]. In the United Kingdom (UK), the National Institute for Health and Care Excellence (NICE) has produced a public health guideline and quality standard for domestic violence with recommendations on: service planning; commissioning; multi-agency working; identifying abuse; specialist advocacy and support for victims and perpetrators; and training for professionals [9,10].

Older people are the fastest growing subset of people worldwide, with those aged over 60 years expected to triple from 672 million in 2005 to nearly 1.9 billion by 2050 [11]. In light of this ageing population, the prevalence and incidence of IPV amongst older women is likely to increase. It is, however, important to make a distinction between IPV and elder abuse, which is defined as "a single, or repeated act, or lack of appropriate action, occurring within *any* relationship where there is an expectation of trust, which causes harm or distress to an older person" (our emphasis). Elder abuse can take various forms such as financial, physical, psychological, sexual, and intentional or unintentional neglect [12].

It is crucial to understand how older women experience IPV (which may be different to younger women) in order to better identify IPV and plan targeted support services. Older women may have different barriers to seeking support due to ill health or dependency on the perpetrator for care or income. Taking a life-course approach, there may be times in a woman’s life where she becomes more susceptible to abuse such as perimenopause, retirement, or when children leave home. Older women may also be less aware of available support.

We present a narrative review of literature on IPV in older women. Our specific objectives are to synthesise data on: (1) prevalence of IPV; (2) associated factors; (3) impact of IPV; (4) responses to IPV; (5) IPV interventions; and (6) key populations. We aim to identify gaps in evidence, highlight research priorities and discuss implications for practice.

## 2 Methods

### 2.1 Data extraction

All authors undertook a systematic search of the following electronic library databases in August 2018: PubMed, Embase, CINAHL and PsycINFO. We aimed to identify original research papers examining IPV in older women (therefore excluding domestic violence perpetrated by other family members, and elder abuse where the perpetrator was not a partner). In this review, we define older women as those aged ≥45 years, in order to capture studies focusing on midlife and the effects of the menopause on IPV risk.

The search strategy was developed by all authors and informed by a 2015 Cochrane Review on IPV screening [13]. Search terms included: battered women, domestic violence, DV, domestic abuse, spous* abuse, intimate partner violence, IPV, old*, midlife, mid-life, middle-aged, elder*, geriatric, climacteric, menopaus*, over 45, 45 and over, wom*n and female*. We applied these search terms to titles/abstracts in all databases, and restricted all searches to studies in English. We did not restrict by date.

We selected studies in women aged ≥45 years (or where disaggregated data were presented in women aged ≥45 years). Studies including both men and women were considered if gender-disaggregated data were presented. Our inclusion criteria included cis *and* transgender women, however no studies in older transgender women were identified, revealing an important research gap. Quantitative, qualitative and intervention studies were all eligible for inclusion. Final selection was discussed by all authors and consensus reached.

Our initial database search identified 414 abstracts, reduced to 400 after removing duplicates. On reviewing abstracts, 83 were selected for full text review, of which 48 met our inclusion criteria and are included in this review.

## 3 Results

### 3.1 Prevalence of IPV in older women

Comparing prevalence estimates across studies is challenging due to differences in definitions of IPV, and time cut offs for current IPV (Table 1). Estimates of lifetime prevalence of all forms of IPV in women aged ≥45 years ranges from 16.5%–54.5% [[14], [15], [16], [17], [18], [19], [20], [21], [22], [23]], with current IPV (defined as within 6–24 months depending on study) rates ranging from 2.0%–28.5% [17,18,20,[24], [25], [26], [27], [28], [29], [30]]. There is a clear trend in the literature towards psychological IPV (either current or lifetime) being more commonly reported than either physical or sexual IPV; a recent multicentre study across Albania, Brazil, Canada and Colombia found a lifetime prevalence of psychological IPV of over 40% [14]. The same study demonstrates a difference in prevalence of current IPV across countries with Brazilian respondents reporting the highest levels of psychological IPV, and Albanian respondents reporting the highest levels of physical IPV [14]. The majority of studies find that IPV prevalence declines with age [15,21,24,27,29]. However, when forms of IPV are looked at separately it appears that whereas physical IPV declines, rates of non-physical forms of abuse remain similar across age groups [24,28,29]. Data comparing prevalence of IPV in older men and women are scant and contradictory; one study of Chinese migrants in the USA reports no difference whilst a Canadian study reports a higher lifetime IPV prevalence in women [18,20].

**Table 1:** Prevalence of intimate partner violence (IPV) in older women.

Reference	Aims	Sample	Design	Prevalence
[14]	To examine associations between lifelong domestic violence and frailty in four countries.	N = 2002 (1047 women) Aged 65-74 Albania, Brazil, Canada, Colombia	Cross-sectional analysis of data from the International Mobility in Aging Study (IMIAS), a population-based prospective study.	- Psychological IPV ever: 40.2%. - Physical IPV ever: 13.1%.
[15]	To compare the health of women with history of (i) physical IPV; (ii) sexual IPV; (iii) physical and sexual IPV; and (iv) no IPV.	N = 3008 women (1170 with history of physical and/or sexual IPV) Women enrolled in a health plan Aged 18-64 USA	Cross-sectional questionnaire study.	- Age 45-54: 37.7% physical IPV, 32.6% sexual IPV, 37.3% physical and sexual IPV (all ever). - Age 55-64: 28.5% physical IPV, 26.5% sexual IPV, 31.7% physical and sexual IPV (all ever).
[16]	To determine prevalence of domestic violence in women attending a gynaecology outpatient clinic and its association with gynaecological symptoms.	N = 825 women 183 aged 50+ Women attending UK gynaecological outpatients	Cross-sectional questionnaire study.	- Age 51-60: 6.8% physical domestic violence ever. - Age >60: 7.6% physical domestic violence ever.
[17]	To identify lifetime and current IPV among midlife women.	N = 354 women Aged 51-62 Australia	Cross-sectional data from a longitudinal study.	- IPV ever: 28.5%. - Physical IPV in past 12 months: 5.5%. - Sexual IPV in past 12 months: 11.8%.
[18]	To examine correlates of current and past violence by intimate partner and family members in community-dwelling senior.	N = 799 (422 women) Aged 65-74 Canada	Cross-sectional analysis of longitudinal data from IMIAS.	- Psychological IPV in past 6 months: 17.8%. - Physical IPV in past 6 months: 0%. - Psychological IPV ever: 16.6%. - Physical IPV ever: 7.1%.
[19]	To examine the association of domestic violence with physical and mental health of older women.	N = 277 women Aged 50-79 USA	Cross-sectional questionnaire study.	- Domestic violence ever: 31.9%.
[20]	To examine experiences and perceptions of domestic violence among older Chinese immigrants.	N = 77 men and women Number of women not reported Aged 50-86 USA	Analysis of cross-sectional data from larger survey of Chinese Americans, disaggregated by gender.	- Minor physical abuse in past 12 months: 7.1%. - Severe physical abuse in past 12 months: 0%. - Minor physical abuse ever: 14.3%. - Severe physical abuse ever: 3.6%.
[21]	To explore prevalence and factors associated with IPV among older women.	N = 10,264 women (2809 aged 50+) Aged 16-86 Germany	Cross-sectional analysis of national survey data.	- Age 50-65: 23.0% IPV ever. - Age 66-86: 10.0% IPV ever.
[22]	To explore unmet domestic violence needs of older women in a clinic population.	N = 110 women Aged 55+ Two mental health centres USA	Cross-sectional questionnaire study.	- Domestic violence ever: 54.5%.
[23]	To examine prevalence of IPV among older women in primary care.	N = 995 women Aged 55+ Registered with primary care USA	Cross-sectional questionnaire study.	- Psychological IPV ever: 2.5%. - Physical IPV ever: 1.6%. - Sexual IPV ever: 2.1%.
[24]	To examine the prevalence of IPV and its association with physical and mental health symptoms in older women.	N = 10,264 women (2809 aged 50+) Aged 16-86 Germany	Cross-sectional analysis of national survey data.	- Age 50-65: 30.0% IPV in past 12 months. - Age 66-86: 27.0% IPV in past 12 months.
[25]	To examine prevalence of IPV and associated factors among midlife and older women attending emergency departments and primary care clinics.	N + 620 women Aged 50-64 Women attending emergency departments or primary care clinics USA	Cross-sectional questionnaire study.	- IPV in past 24months: 5.5%.
[26]	To describe rates of self-reported IPV among older women within a private tertiary women’s health clinic.	N = 1389 women Aged 50-90 Women attending women’s health clinic USA	Cross-sectional questionnaire study.	- Verbal abuse in past 12 months: 7.0%. - Physical abuse in past 12 months: 1.0%. - Sexual abuse in past 12 months: <1.0%.
[27]	To define the extent and nature of IPV among older women.	National sample of women (number not reported) Aged 12+ USA	Analysis of longitudinal data from the National Crime Victimization Survey.	- Age 55+: 2.0% IPV in past 6 months (incidence 0.44/1000).
[28]	To examine the relationship between age, physical violence and non-physical abuse within the context of IPV.	N = 1249 women Aged 18-69 Number in age groups not reported USA	Cross-sectional analysis of data from the Michigan Violence Against Women Survey.	- Age 53-57: 21.8% past year IPV. - Age >58: 25.0% past year IPV.
[29]	To identify the prevalence of past-year IPV among women Veterans and document associated demographic, military, and primary care characteristics.	N = 6287 women Aged 18+ Number in age groups not reported Veterans health Administration (VHA) primary care users USA	Cross-sectional analysis of retrospective cohort data from the Women’s Overall Mental Health Assessment of Needs survey.	- Age 45-54: 22.2% past year IPV. - Age 55-64: 15.8% past year IPV. - Age 65+: 4.9% past year IPV.
[30]	To explore associations between socioeconomics/social relations and domestic violence in older adults.	N = 1209 (525 women) Aged 65-74 Albania, Brazil, Canada, Colombia	Cross-sectional analysis of data from IMIAS.	- Psychological IPV in past 6 months: 18.9%. - Physical IPV in past 6 months: 1.3%.

### 3.2 Factors associated with IPV in older women

Studying factors associated with IPV is similarly challenging due to the lack of consistent definitions and age ranges employed (Table 2). Six cross-sectional survey studies identify the following associations in older women: frailty; inadequate partner support; white ethnicity; special needs or disabilities; alcoholism; obesity; other family violence; recent homelessness; and sexual health risks [14,25,26,30,31]. Alcoholism and other experiences of family violence as associated factors were further supported by qualitative data [18,26,32,33]. Retirement was cited as a key factor in precipitating violence in two studies [33,34]. Other life events identified as precipitating factors for violence included children leaving home, partners experiencing poor health, the menopause, and marriage [[34], [35], [36]]. However, marriage has also been identified as a protective factor [26]. As with IPV affecting younger women, there is an association between IPV and power/control dynamics [34,37].

**Table 2:** Factors associated with intimate partner violence (IPV) in older women.

Reference	Aims	Sample	Design	Associated factors
[14]	To estimate the prevalence of frailty in older adults. To describe the association between frailty and domestic violence including IPV.	N = 2002 (1047 women) Aged 65-74 Albania, Brazil, Canada, Colombia	Cross sectional study based on data from International Mobility in Aging Study.	- Psychological IPV is associated with frailty.
[18]	To identify correlates of current and past violence by intimate partners and family member(s) in community-dwelling Canadian seniors, while accounting for childhood adverse circumstances.	N = 398 (210 women) Aged 65-74 Canada	Cross-sectional study (baseline data from the International Mobility in Aging Study IMIAS).	- Lifetime IPV was associated with: being female, alcohol consumption, obesity and having experienced lifetime family violence from a family member.
[25]	To examine prevalence of and associated factors for IPV among older women attending an emergency department and primary care in an urban setting.	N = 620 Aged 50-64 USA	Cross-sectional study.	- Factors associated with IPV included recent history of homelessness, and HIV seropositivity.
[26]	To report rates of self-reported IPV with a focus on verbal abuse among older women in a private tertiary women’s health clinic.	N = 1389 Aged 50-90 USA	Cross-sectional questionnaire study.	- The following factors were associated with verbal abuse in multivariate analyses: alcoholism and physical abuse. - Marriage appeared to be a protective factor against verbal abuse.
[30]	To describe the association between gender, socioeconomic status and social support structures with domestic violence in older people.	N = 1995 (1040 women) Aged 65-74 Albania, Brazil, Canada, Colombia	Cross sectional study based on data from International Mobility in Aging Study.	- Low and mid-levels of partner support were associated with greater odds of psychological IPV amongst older women.
[31]	To examine the differences between older and younger women who use IPV services.	N = 5,235 2,495 Aged 18-64 2,740 Aged 65+ USA	Cohort study.	- Factors associated with IPV in older women included: White ethnicity, and special needs or disabilities.
[32]	To examine the trajectory of, and community responses to, violence in late life in rural Kentucky.	Focus groups: N = 24 professionals working with women experiencing IPV Individual interviews: N = 10 rural women experiencing IPV Aged 50-69 USA	Qualitative study (focus groups & in-depth interviews).	- Associated factors included: living in multiple abusive households since childhood, and drug and alcohol use by the perpetrator enhancing the violence.
[33]	To examine the experiences of women aged fifty and older who had experienced IPV.	N = 64 Aged 50-87 Canada	Qualitative action-research study.	- Associated factors included: having a history of previous abusive relationships and retirement.
[34]	To explore the experiences of psychological violence perpetrated by a partner amongst older women.	N = 15 Aged 60-81 Canada	Qualitative semi-structured interviews.	- Control dynamics of IPV increased at retirement, when children left home and when partners experienced poorer health.
[35]	To explore the attitudes to, and experience of menopause among Macedonian women.	N = 81 Aged 45-75 Australia	Qualitative study (unstructured group discussions).	- Shift in male attitudes to regarding women as “non-sexual” around the menopause was seen as a precipitating factor in domestic violence.
[36]	To describe the experiences of older women who participated in a hospital-based domestic violence intervention program.	N = 4 Aged 63-73 Canada	Qualitative interview study.	- Marriage being viewed as a licence to abuse.
[37]	To capture women’s perspectives on the experience of domestic violence in later life.	N = 135 Aged 45-85 18 “non-victim” focus groups 3 “known-victim” focus groups USA	Qualitative focus group study (Domestic Violence Against Older Women study).	- Power and control was identified as a major theme underlying abuse and sub-themes included deep-seated inclination to be submissive, low self-esteem, and belief in the sanctity of marriage.

### 3.3 Impact of IPV on older women

It is clear that IPV has significant health and psychosocial consequences in older women, often exacerbated by the duration of exposure to violence (Table 3). Current and lifetime IPV is associated with an increased likelihood of physical symptoms including gastrointestinal, respiratory, pelvic and genitourinary symptoms, as well as being associated with frailty [14,24,38,39]. A US study reported an association between IPV and HIV seropositivity, although due to the cross-sectional study design it is difficult to ascertain if HIV infection is a cause or consequence [25]. The same authors report an association between current IPV and both condomless sex and sex with a partner at high-risk of HIV, suggesting challenges in negotiating safer sex [25].

**Table 3:** Impact of intimate partner violence (IPV) on older women.

Reference	Aims	Sample	Design	Prevalence
[14]	To examine associations between lifelong domestic violence and frailty in four countries.	N = 2002 (1047 women) Aged 65-74 Albania, Brazil, Canada, Colombia	Cross-sectional analysis of data from the International Mobility in Aging Study (IMIAS), a population-based prospective study.	- - Psychological IPV ever associated with frailty (AOR 2.11;1.36,3.26). - Physical IPV ever not associated with frailty.
[24]	To examine the prevalence of IPV and its association with physical and mental health symptoms in older women.	N = 10,264 women (2809 aged 50+) Aged 16-86 Germany	Cross-sectional analysis of national survey data.	- IPV in past 12 months associated with increased odds of gastrointestinal symptoms, pelvic problems and psychological symptoms in women of all ages but effect stronger in those aged 66-86.
[25]	To examine prevalence of IPV and associated factors among midlife and older women attending emergency departments and primary care clinics.	N = 620 women Aged 50-64 Women attending emergency departments or primary care clinics USA	Cross-sectional questionnaire study.	- IPV in past 24 months associated with condomless sex with partner and sex with partner at risk of HIV. - IPV in past 24 months associated with sexually transmitted infection in last 6 months and HIV seropositivity.
[33]	To describe the experiences of older women with a history of abuse.	N = 64 women with history of abuse Aged 50+ Includes abuse by partners and children Canada	Qualitative interview study	- Abuse throughout married life. - Impact of illness e.g. partners resenting caring duties.
[37]	To explore experiences of nonphysical IPV among middle-aged and older women.	N = 134 women (including women with experience of IPV) Aged 45-85 USA	Qualitative focus group study.	- Long-term systematic destruction of self-worth and self-efficacy. - Isolation from friends and family.
[38]	To investigate the association between domestic violence and physical health in midlife Australian women.	N = 14,100 Aged 45-50 Australia	Cross-sectional analysis of Australian Longitudinal Study on Women’s Health.	- Domestic violence ever associated with increased odds of current gastrointestinal, respiratory, genitourinary and pain symptoms.
[39]	To understand how older women cope with domestic violence and how it affects their wellbeing.	N = 18 women who had been in abusive relationship since aged 50+ Aged 53-72 UK	Qualitative interview study.	- Prolonged lifelong effects of abuse. - Impact on health: chronic pain, fatigue, arthritis, hypertension, mental health. - Guilt at not leaving earlier. - Long-term isolation leading to profound dependency.
[40]	To explore experiences of violence among young and old battered women.	N = 40 battered women (17 aged 60-84) Aged 23-84 Israel	Qualitative interview study.	- Isolation over numerous years. - Bodily pain exacerbated by ageing – signs of violence may be misattributed to age. - Loss of control over body and home. - Loneliness if leaves partner due to loss of others in social network. - Accumulated wisdom and coping strategies.
[41]	To examine the psychological health correlates of domestic violence in midlife Australian women.	N = 11,310 Aged 47-52 Australia	Cross-sectional analysis of Australian Longitudinal Study on Women’s Health.	- Domestic violence ever associated with history of depression (AOR 1.93;1.70,2.19) and anxiety (AOR 1.97;1.72, 2.26), and current medication use for depression (AOR 1.66; 1.36,2.02) or anxiety (AOR 1.49;1.18,1.87).
[42]	To explore the impact of domestic abuse on the health and lives of older women.	N = 16 women who had experienced domestic abuse Aged 63-79 UK	Qualitative interview study.	- Impact on health in context of ageing body. - Mental health impacts e.g. anxiety. - Impact on relationships with children who grew up in abusive home impacting on support in later life.
[43]	To describe and analyse the experiences and perceptions of older battered women.	N = 20 battered women Aged 66-80 Israel	Qualitative interview study.	- Framing of self on spectrum from ‘foolish victim’ to ‘heroic survivor’. - Abandoning of self for others e.g. children. - Unfulfilled expectations and hope, exacerbated when children leave home. - Notion of past as a burden and future as unclear.
[44]	To explore experiences of loneliness among older battered women.	N = 21 battered women Aged 60-85 Israel	Qualitative interview study.	- Enhanced sense of loneliness and social isolation over many years of abuse. - Separation from children to ‘save them’ from abuse.
[45]	To describe the experiences of older women diagnosed with breast cancer experiencing IPV.	N = 11 women with breast cancer and experiencing IPV Aged 51-84 USA	Qualitative interview study.	- Partners exerting control over management e.g. attending appointments, controlling treatment, insisting on wig wearing. - Isolation leading to limited support during cancer treatment - Women physically and mentally less able to cope with IPV.

AOR, adjusted odds ratio.

As women get older there is a risk that the physical consequences of IPV may be attributed to age-related illness [40]. IPV also adversely impacts older women’s psychological wellbeing; women reporting current or lifetime IPV are more likely to have poor mental health (such as depression and anxiety), with one study finding that experiencing IPV in the past 12 months was associated with four times the odds of psychological symptoms [24,41,42].

Qualitative research on experiences of IPV in older women reveals the considerable impact of prolonged abuse, and how pivotal social transitions such as children leaving home can lead to feelings of hopelessness and unfulfilled expectations of life [43]. Women describe profound isolation, exacerbated by years of physical and psychological violence, leading to dependency on abusive partners which is increased by fears of ageing. This isolation often extends to children, with women often trying to protect children from violence by creating distance [42,44]. The shrinking of social networks is likely to be of particular concern as women get older and require more in the way of social support.

One study, exploring IPV in older women diagnosed with breast cancer, highlights the particular challenges faced by this group including loss of control over treatment decisions, and the impact of physical symptoms on ability to cope with IPV [45]. Furthermore, illness may also precipitate or exacerbate IPV as a result of partners’ resentment of new caring roles [33].

### 3.4 Responses to IPV among older women

Help-seeking amongst older women experiencing IPV is complex with women describing numerous barriers (Table 4). Almost all studies identified responses from others as a key influence on women’s help-seeking behaviours [32,33,36,37,39,42,[45], [46], [47], [48], [49]]. These include the response of professional services such as law enforcement, legal and health agencies, specialist IPV services, housing and welfare services, family members, friends, and community organisations. Specific barriers to help-seeking include lack of trust, knowledge, action, and emotional support. These barriers are further exacerbated by negative self-perception and internal feelings of guilt, shame, embarrassment, and powerlessness [32,36,39,46,50].

**Table 4:** Responses to intimate partner violence (IPV) among older women.

Reference	Aims	Sample	Design	Responses
[32]	To examine the trajectory of, and community responses to, violence in late life in rural Kentucky.	Focus groups: N = 24 professionals working with women experiencing IPV Individual interviews: N = 10 rural women experiencing IPV Aged 50-69 USA	Qualitative study (focus groups & in-depth interviews).	- Barriers to leaving: control tactics and diminished feeling of self-worth; providing for children/grand-children; belief that the abuse might stop; lack of personal resources; acceptance of the abuse; protective orders not enforced; lack of community support; difficulty with rehousing; and community networks. - Facilitators to leaving: threat to their own or their children’s lives.
[33]	To examine the experiences of women aged fifty and older who had experienced IPV.	N = 64 Aged 50-87 Canada	Qualitative action-research study.	- Barriers to help-seeking or leaving included: difficulty accessing safe housing; healthcare requirements; financial difficulties; difficulty finding a job; language restrictions; and lack of help from religious leaders. - Women reported a number of different sources of help when leaving an abusive situation including shelters/shelter staff, family physicians, and daughters.
[36]	To describe the experiences of older women who participated in a hospital-based domestic violence intervention program.	N = 4 Aged 63-73 Canada	Qualitative interview study.	- 11 themes identified in total. - 2 related to barriers in seeking help: a feeling of powerlessness and specific barriers to leaving (inability to obtain divorce, lack of resources, age, and effect on children). - 2 related to facilitators to seeking help: community support as pre-cursor and turning points (severe violence, support from health providers).
[37]	To capture women’s perspectives on the experience of non-physical domestic violence in later life.	N = 135 Aged 45-85 18 “non-victim” focus groups 3 “known-victim” focus groups USA	Qualitative focus group study (Domestic Violence Against Older Women study).	- Barriers to help-seeking included invisibility of non-physical abuse and concerns about community and law enforcement responses.
[39]	To understand how older women cope with domestic violence and how it impacts their wellbeing.	N = 18 Aged 53-72 UK	Qualitative semi-structured interview study.	- Multiple barriers to help-seeking were identified including: limited support from family and friends including their children; response from legal, health and social care professionals; lack of community resources; limited knowledge of the criminal justice system; economic dependence on the perpetrator; caring for the perpetrator; and shame, guilt, and self-blame.
[42]	To explore the impact of domestic abuse on the health and lives of older women.	N = 16 Aged 63-79 UK	Qualitative in-depth interview study.	- Potential barriers to reporting included shame, embarrassment, absence of formal/informal support networks, a lack of “permission” to speak out, and that services would not be able to meet their particular needs as older women.
[43]	To describe and analyse the experiences and perceptions of older Jewish women in coping with domestic violence.	N = 20 Aged 60-80 Israel	Qualitative interview study.	- Giving up the self for the sake of family members as a barrier to help-seeking.
[45]	To describe the experiences of older women diagnosed with breast cancer while experiencing IPV.	N = 11 Aged 51-84 USA	Qualitative semi-structured interview study.	- Coping mechanisms included: accessing support (support groups, children and grandchildren, other family members, and church communities) and personal strategies (journaling, seeking personal counselling, exercise, and diet).
[46]	To identify and explore the needs of older and isolated women who lives with domestic violence.	N = 90 Aged 50-78 Australia	Mixed methods qualitative study (face to face interviews, focus groups, national phone-in).	- 5 barriers to leaving: lack of knowledge about domestic violence; matters involving disability (their own, their partners, caring for family members); attitudes and values (feelings of shame, embarrassment, loyalty to family); caring for others (parents, animals, gardens); and grief about their lives and their children’s lives.
[47]	To identify opportunities and challenges in promoting community support for rural older women experiencing IPV.	N = 72 service providers, 10 women with experience of IPV Aged 54-70 USA	Mixed methods community-based participatory research study: literature search cross-sectional survey, focus groups, individual interviews, workshops.	- Key findings from service providers included limited awareness about IPV in older women including services available and stereotyped notions of associations. - Key findings amongst older women who had experience IPV included resisting help until violence was life-threatening as well as a need for discreet information about service, improved professional sensitivity and more appropriate housing.
[48]	To understand and identify what women aged 50 and older want and need from domestic violence services.	N = 28 Aged 50+_ Personal experience of IPV was not in the inclusion criteria USA	Qualitative focus group study.	- 5 major themes contributing to help-seeking: importance of family and friends; trust in physicians and mitigated trust in ministers; interest in understanding law enforcement; importance of language in outreach and intervention; appropriate outreach and services.
[49]	To explore the experiences of and response to abuse and neglect among older immigrant women in the Sri Lankan Tamil community.	N = 43 Aged 48-85 Canada	Qualitative individual interviews & focus groups.	- 6 themes related to barriers to seeking help were identified: children’s and grandchildren’s welfare; family expectations; community expectations; unfamiliarity with the new setting (transportation, language); limitations in availability, accessibility and appropriateness of formal social support; and financial and immigration concerns.
[50]	To describe a model of barriers to seeking help among older women experiencing domestic violence.	N = 134 Aged 45-85 USA	Qualitative focus group study: *Domestic Violence Against Older Women* (DVAOW) Study.	- 12 interrelated themes based on 3 categories of barriers to help-seeking: - Abuser behaviours – isolation; intimidation; jealousy - Internal factors - protecting family; self-blame; powerlessness; hopelessness; secrecy - External factors - family response; clergy response; justice system response; community responsiveness.
[51]	To describe types of abuse affecting older women and how they characterize the abuser.	N = 38 Aged 55-90 USA	Qualitative semi-structured interview study.	- Women used labels to “make sense” of the abuse including: mental health illnesses, drug and alcohol problems, ethnic stereotyping, underlying homosexuality, “women-hating”, and narcissistic personality traits.
[52]	To understand how older victims of IPV have coped or current cope.	N = 38 Aged 55-90 USA	Qualitative interview study.	- Reported a mix of problem-focused coping behaviours and emotion-focused coping behaviours with the latter being favored. - Specific themes included: reappraisal or reframing aspects of their situation; reorientation (refocusing role in the family and reaching out to others); and maintaining the appearance of conjugal unity.

Financial dependence on the perpetrator is another key barrier to help-seeking [32,33,36,39,49]. One study highlighted that this is amplified by the difficulties older women encounter in finding employment [33]. Women’s characterisation of perpetrators also influenced decisions to stay; women used labels relating to mental health problems, narcissistic and “women-hating” personality traits, ethnic stereotyping, underlying homosexuality, and drug and alcohol misuse as ways of explaining and coping with the abuse [51].

Family plays a critical role in women’s responses to IPV, especially in choosing to stay or leave an abusive situation. Protecting or caring for family members, particularly children, are frequently identified as reasons for staying in an abusive situation. In one study, the caring role of older women in families as a barrier to leaving extended to caring for the perpetrator [32,36,39,43,46,49,50,52]. However, the protective role of women towards children had another side. One study identified a threat to children’s lives as a critical lever for leaving an abusive situation [32]. Two studies identified family support, including from children, as a key factor in coping with IPV and seeking help [33,45].

### 3.5 IPV interventions for older women

There are no randomised controlled trials of interventions for older women experiencing IPV, and only four observational studies describe the use of IPV services amongst older women [31,[53], [54], [55]]. In one study, older people represented 2.5% of all specialist IPV service users [53]. In another study, use of services by older women differed from younger women; older women were more likely to use advocacy and legal services, but less likely to use others including housing, financial, employment, case management, substance abuse, and counseling services [31]. One US study identified that service use was limited amongst older women due to staff education and poor inter-agency collaboration [55]. Similar findings were identified through qualitative methods in a further US study [56] and a lack of dedicated services for older women was highlighted in another [57]. These problems could potentially be addressed by government recommendations; in one study, state recommendations resulted in an increase in provision of tailored IPV services for older women within 5 years [54].

Data on the effectiveness of IPV interventions for older women is limited to qualitative studies [58,59]. Support groups were viewed as a good opportunity to make life changes [58]. This study used interviews with professionals working with IPV victims to identify characteristics of effective support groups. Key characteristics included: being open access; culturally-specific; and all-female with mixed locations and timings to promote access. However the same study identified facilitating participation, obtaining funding, and lack of transport or freedom to attend as major challenges faced by support groups [58]. An analysis of case studies described effective services as welcoming, engaging, encouraging, supportive, and empowering [59]. Service design should ideally reflect service user voices. One study summarises what older women want from services: being believed; social support; access to information; appropriate responses from professionals; accurate legal information and services; income support; and help with accommodation [46]. Women reported using informal and formal resources, and would welcome greater access to all [60] (Table 5).

**Table 5:** Intimate partner violence (IPV) interventions for older women.

Reference	Aims	Sample	Design	Intervention Findings
[31]	To examine the differences between older and younger women who use IPV services.	N = 5,235 2,495 Aged 18-64 2,740 Aged 65 and over USA	Cohort study.	- Older women were more likely to use civil and/or criminal advocacy services related to obtaining orders of protection. - Older women were less likely to use the majority of other services. - Older women were more likely to obtain help via a police referral or a State’s Attorney’s office. - Older women were less likely to seek help via a friend or a self-referral.
[46]	To identify and explore the needs of older and isolated women who lives with domestic violence.	N = 90 Aged 50-78 Australia	Mixed methods qualitative study (face to face interviews, focus groups, national phone-in).	- Needs identified related to the following themes: being believed; having social support; accessing tailored information; appropriate responses from service providers (healthcare providers, ministers of religion; legal professionals; and the police); accurate and accessible legal support; income support; and availability and suitability of accommodation.
[53]	To describe the variation in use of services provided by the Illinois Coalition Against Domestic Violence amongst older women of different ethnicities between 1990 & 1995.	N = 2702 Aged 55+ USA	Retrospective cohort study.	- Older people represented 2.5% of all service users.
[54]	To describe the impact of state recommendations for special programming for abused older women in Florida.	N = 33 shelters USA	Pre- and post-survey study.	- State recommendations resulted in better provision of IPV services for older women: more shelters and more staff, volunteers, and board directors aged over 60.
[55]	To describe the domestic violence services in Ohio in relation to women aged 55 and over.	N = 52 Domestic violence centres USA	Cross-sectional statewide survey study.	- For women aged 55+: 36% provided outreach services; 29.8% had provided direct services or a referral to at least one woman; 19.6% had served at least one woman via a crisis line; 40.1% had served at least one woman via a support group. - Areas for improvement: education; training; collaboration with aging agencies
[56]	To describe community professionals’ awareness, perceptions and experience in providing support to older women experiencing intimate partner violence.	N = 87 Community professionals likely to encounter women experiencing IPV USA	Qualitative focus group study.	- Most professionals were unaware of the extent of the problem. - Support was provided in services with little collaboration unless there was also a health emergency.
[57]	To explore service responses to abuse among older people across a range of sectors.	N = 18 agency workers N = 3 older women	Mixed methods study (questionnaires & in depth interviews).	- 3 main themes: - Lack of conceptual clarity between domestic abuse and elder abuse - Complexity of family dynamics and abusive relationships. - Deficit in dedicated service provision for older survivors.
[58]	To describe the role of support groups for older women experiencing domestic violence.	N = 34 Support group facilitators USA	Qualitative interview study.	- Main benefit of support groups is the opportunity and information for women to make changes to their lives. - Characteristics of existing support groups include: being open access and culturally specific; mostly all-female; address historical and current abuse; having mixed locations such as DV agencies, health settings, community settings; occurring at varied times of days; and content varying between informal meetings, peer counselling and structured activities to address specific problems. - Challenges to starting support groups include facilitating participation, lack of transportation to attend, freedom to leave when living with an abuser, and funding.
[59]	To understand the most effective interventions when working with older women who have experienced IPV.	N = 2 Aged 63-65 USA	Qualitative study (case studies).	- Themes identified for effective interventions: welcoming and engaging the client; encouraging and supporting the telling of one’s story; and assisting in the process of empowerment.
[60]	To provide insight into resources used to leave an abusive partner.	N = 8 Aged 50-74 Canada	Qualitative semi-structured interview study.	- Women used formal and informal resources. - Formal resources included family members, friends, neighbours, and self-help. - Informal resources included criminal justice system, financial resources, mental health services, family violence services, physical health services, housing services, transportation services, addiction services, employment and volunteering services, security and communication services, and educational programs. - Greater access to all resources would have been welcomed by the women.

### 3.6 IPV in older women from key populations

Only six studies looked at IPV experienced by older women in key populations, demonstrating a paucity of data in this area (Table 6).

**Table 6:** Intimate partner violence (IPV) in older women from key populations.

Reference	Aims	Sample	Design	Findings
[20]	To examine experiences and perceptions of domestic violence among older Chinese immigrants.	N = 77 men and women Number of women not reported Aged 50-86 USA	Analysis of cross-sectional data from larger survey of Chinese Americans, disaggregated by gender.	- 7.1% of women had experienced minor physical violence by their spouses in the last 12 months, 14.3% women had lifetime experience of minor physical violence. - Gender and acculturation were associated with perceptions and attitudes towards domestic violence.
[29]	To identify the prevalence of past-year IPV amongst women veterans utilizing the Veterans Health Administration (VHA) primary care, and to identify the associated demographic, military and primary care characteristics.	N = 6287 women veterans Aged 18-65+ (exact age range not given). USA	Retrospective cohort based on a telephone survey linked to administrative data in the year prior to the survey.	- Past year prevalence of IPV 18.5%. - Higher rates in younger women aged up to 55. - Associated factors include economic hardship, lesbian/bisexual, parent/guardian of a child aged <18, experiences of military sexual trauma, <10 years of service. - Women experiencing IPV had more primary care visits, but lower continuity of care across providers.
[46]	To identify and explore the needs of older and isolated women who live with domestic violence.	N = 90 Aged 50-78 Australia	Mixed methods qualitative study (face to face interviews, focus groups, national phone-in).	- Issues of particular relevance to women from rural and remote areas include: geographical isolation and lack of transport; conservative and patriarchal rural culture with women being expected to live and cope with adversity and to overcome all difficulties without complaining or giving in; large numbers of licensed and unlicensed guns, leading to more violent abuse; too few resources/ services such as medical, housing, leisure; “small town syndrome” resulting in a lack of confidentiality; and poverty associated with high unemployment levels.
[47]	To identify opportunities and challenges in promoting community support for rural older women experiencing IPV.	N = 72 service providers, 10 women with experience of IPV Aged 54-70 USA	Mixed methods community-based participatory research study: literature search cross-sectional survey, focus groups, individual interviews, workshops.	- Key findings from service providers included limited awareness about IPV in older women including services available and stereotyped notions of associated factors. - Key findings amongst older women who had experienced IPV included underreporting; resisting help until violence was life-threatening as well as a need for discreet information about service; improved professional sensitivity and more appropriate housing options; cycle of violence - many women had experienced family violence early in their lives.
[49]	To explore older immigrant women’s experiences of and responses to abuse and neglect in one community.	N = 43 Sri Lankan immigrant women Aged 48-85 Canada	Qualitative study (focus groups & interviews).	- Older women experienced various forms of neglect and abuse and their primary abusers were husbands, children and children in law. - Their community and Canadian society at large contributed to experiences of abuse. - Women’s responses to abuse were shaped by multi-level factors. - In responding to abuse, older immigrant women showed remarkable resilience.
[61]	To describe the types of IPV and sexual HIV-risk factors reported by the sample and to provide estimates of the associations between experiencing IPV in a primary heterosexual relationship and HIV-risk indicators.	N = 139 African American and Latina women Aged 50-83 USA	Cross-sectional survey.	- Older women who experience IPV are at elevated risk for HIV. - Factors associated with IPV were multiple partners within the last year, having a primary partner engaging in HIV risk behaviours/HIV positive. - Women with multiple sexual partners were more likely to report lifetime IPV. perpetrated by their primary partners - Women in relationships with partners with known HIV risk were more likely to report lifetime IPV.

#### 3.6.1 Rural settings

Two studies looked at IPV in older women who lived in rural settings in Australia and the US [46,47]. They found that living in a rural community was a barrier to seeking support due to geographical isolation, economic deprivation and a lack of social services. As these communities are often small, fear about the lack of confidentiality deterred women from telling others about their abusive relationships. Conservative and patriarchal cultural norms within these communities often meant that women were expected to cope with adversity on their own. The ready availability of guns in these settings meant that violence was often more severe. Women commonly resisted seeking help until the violence was life-threatening.

#### 3.6.2 Post-migration contexts

Two studies addressed older women in the post-migration context and found that vulnerability to abuse and neglect often increased after migration due to: geographical and social isolation; change in family dynamics; language difficulties; and financial and transport dependency. Barriers to accessing support included a lack of knowledge of services and culturally specific services, pressure from the wider migrant community, and immigration laws often leaving them dependent on a spouse or child for residency status. A study of older Chinese migrants found that those who were less acculturated were less likely to define physical violence towards a woman as domestic violence and more likely to think the violence was justified [20]. A study of older Sri Lankan women who had moved to Canada found that older immigrant women showed remarkable resilience. Many were encouraged to leave their abuser as a result of having migrated to Canada [49].

#### 3.6.3 HIV risk in women of colour

One study looking at African American and Latina women of colour in the US aged ≥50 years found that those who had experienced IPV were more likely to report HIV-risk behaviours such as: condomless sex; having more than one partner in the previous year; and a primary partner with known HIV risk. They also had a poorer perception of their own HIV risk than those who hadn’t experienced IPV, despite more than a quarter having had a previous sexually transmitted infection [61].

#### 3.6.4 Veterans

One study looked at women veterans accessing primary care in the US and found that experiences of military sexual trauma were associated with IPV. Those experiencing violence visited clinics more often, although continuity was fractured across primary care providers [29].

## 4 Conclusions

This review synthesises existing data on IPV in older women in order to identify gaps in evidence and make recommendations for practice and research.

We found that IPV in women aged ≥45 years is common, with psychological abuse being the most commonly reported form. The majority of studies found that IPV prevalence declined with age. This was driven by declining physical violence; other types of abuse remained similar across age groups. Factors associated with IPV in older women include frailty, inadequate partner support, special needs or disabilities, alcoholism, obesity, other family violence, recent homelessness and sexual health risk taking. Precipitating factors for IPV for older women in particular have been identified such as retirement, children leaving home, menopause, and poor health of partners. These are significant transition times for older women and may parallel the link seen in younger women between IPV and pregnancy, another significant transition time in a woman’s life course [62].

Similar to younger women, IPV has significant health and psychological impacts on older women, often exacerbated by the duration of exposure to violence. Prolonged abuse and pivotal social transitions such as children leaving home can lead to feelings of hopelessness, unfulfilled life expectations and profound social isolation. Physical and psychological symptoms are also reported to be similar to young women, but there is also an additional association with frailty in older women. Increasing illness and frailty mean that women may need to depend more on their partner, which can lead to increased violence.

The reasons why older women experiencing IPV may not access help are complex. Barriers include a lack of knowledge or trust in services, concerns about the response of others, and financial dependence on the perpetrator. We did not find any randomised controlled trials of interventions for older women experiencing IPV in our review, identifying only four observational studies.

This review highlights a lack of consistency in defining older women in terms of age, and in terminology used to define violence and time cut off for current IPV. Most studies were conducted in North America so may not be generalisable to other settings. It was also difficult to infer causality as all quantitative studies were cross-sectional in design. There were no randomised controlled intervention studies. There were also very little data on key populations, and most notably an absence of data on older transwomen.

It is therefore clear that research priorities include standardisation of definitions including type of violence, older women, and time cut-offs for current abuse. More robust data are also required in the form of longitudinal studies and randomised controlled intervention studies. Furthermore, we call for more work on older women and IPV explicitly focusing on key populations such as women from diverse ethnic groups, transwomen, women with disabilities, women living with HIV, migrant women, and women in same-sex relationships.

With regards to practice, we recommend that services are specifically tailored to older women ensuring they feel believed and emotionally supported, and have access to legal, financial and housing advice. Staff working in non-VAW organisations who may come into regular contact with older women, such as healthcare and social services, should be offered training on how IPV may affect women in this age group. Recent guidance from the British HIV association states that pre-exposure prophylaxis (PrEP) should be considered for women experiencing IPV; health professionals should be aware of the risk of IPV in older women and consider PrEP if IPV is identified [63].

Governments also need to take leadership, recognising the issue and increasing its visibility. Of 131 government reports on violence against women, only 13 recognised that older women were also at risk of violence; a 2013 United Nations report found that domestic violence legislation does not, in general, specifically include older women [64,65].

In conclusion, we found that IPV is common in older women aged ≥ 45 years, and that their age and life transitions mean that they may experience abuse differently compared to younger women. They face unique barriers to accessing help, and services should be tailored to meet their needs.

## Contributors

All three authors were responsible for the conceptualization and methodology of the review, analysis of the data, and drafting and editing the manuscript.

## Conflict of interest

Neha Pathak has previously received WHO funding to attend and present at WHO training on gender-based violence.

Rageshri Dhairyawan has previously received travel bursaries from MSD, ViiV and Gilead Sciences, speaker honoraria and consultancy fees from Gilead Sciences and Janssen - Cilag.

Shema Tariq has previously received a travel bursary funded by Janssen-Cilag through the British HIV Association, speaker honoraria and funding for preparation of educational materials from Gilead Sciences, and is a member of the steering group of SWIFT, a networking group for people involved in research in HIV and women, funded by Bristol Myers Squibb.

## Funding

Neha Pathak is funded by a UCL Wellcome Trust Clinical Research Training Fellowship (Grant Reference 211162/Z/18/Z). Shema Tariq is funded by a UCL Wellcome Institutional Strategic Support Fund Flexible Support Award (Grant Reference 204841/Z/16/Z). Any views expressed in this paper are those of the authors, and not necessarily those of funders.

## Ethical Statement

This research did not involve any primary research on humans or animals. As such, ethical approval was not required.

## Provenance and peer review

This article has undergone peer review.

## CRediT author statement

All authors: Conceptualization, Methodology, Validation, Formal Analysis, Investigation, Resources, Writing – Original Draft, Writing – Review & Editing, Visualization.
